# No Little Feet: Managing Pseudocyesis in a Homeless, Acutely Manic Patient with Schizoaffective Disorder, Bipolar Type

**DOI:** 10.1155/2023/2504871

**Published:** 2023-12-13

**Authors:** Talitha West, Omotola Ajibade, Anthony Fontanetta, Samreen Munir

**Affiliations:** ^1^Department of Psychiatry, Ocean University Medical Center, 1610 Route 88, Brick, NJ 08724, USA; ^2^Forensic Psychiatry, Emory University, 12 Executive Park Dr. NE, Atlanta, GA 30329, USA; ^3^Department of Psychiatry, Hackensack Meridian School of Medicine, 340 Kingsland St., Nutley, NJ 07110, USA; ^4^Department of Psychiatry, Raritan Bay Medical Center, 530 New Brunswick Ave., Perth Amboy, NJ 08861, USA

## Abstract

Pseudocyesis is a complex psychiatric manifestation of the physical symptoms of pregnancy. Although not pregnant, the pseudocyetic patient displays signs and symptoms consistent with pregnancy, such as abdominal distention, cramping, and/or sensations of fetal movement. Pseudocyesis is more common in developing countries than in the developed world, possibly due to the importance that traditional societies attach to childbearing and the low social status that these societies assign to women who are unable to produce children. Socioeconomically disadvantaged women in developed countries may also be at increased risk. Although the etiology, pathogenesis, diagnosis, and management of pseudocyesis are poorly understood, it manifests with real symptoms, which may complicate both the patient's perspective about her condition and the medical and psychiatric teams' approach to the patient. This case report is one of only a few in the literature to present an example of pseudocyesis developing in the context of acute mania. After describing the patient's clinical course, from her initial symptoms of pseudocyesis to their eventual resolution, this report will provide recommendations for the sensitive care of patients with this rare but significant condition.

## 1. Introduction

Pseudocyesis is a rare condition that the Diagnostic and Statistical Manual of Mental Disorders, Fifth Edition (DSM-5) defines as “a false belief of being pregnant that is associated with objective signs and reported symptoms of pregnancy” [1]. While robust epidemiologic data on pseudocyesis are absent, a 2017 narrative review suggests that its incidence in developed nations has declined over the past century, with a reported frequency of 1–6 cases per 22,000 deliveries in the United States in 2007 [2]. The incidence in developing nations is thought to be orders of magnitude higher. For example, a study in a rural Nigerian community estimated its frequency at one in 344 pregnancies [3], and a study of Sudanese women presenting for infertility treatment reported its frequency at 1 in 160 [4]. The condition typically affects women of childbearing age who are experiencing some form of emotional distress in relation to the idea of pregnancy, whether it is ambivalence about pregnancy, fear of pregnancy, or an intense desire to become pregnant despite unsuccessful attempts to conceive [5].

A number of authors have theorized that the condition's higher incidence in developing nations stems from cultural factors that lead women to believe that their inherent value and place in the socioeconomic hierarchy are tied to their childbearing ability [6]. Here, we report on a case of pseudocyesis arising in a patient living in the United States who had been experiencing homelessness for several months and who viewed pregnancy as a source of potential economic gain. The present report adds to the small but growing body of literature describing pseudocyesis in socioeconomically disadvantaged patients living in developed nations, a demographic with which pseudocyesis has not traditionally been associated [5].

Regarding comorbid conditions, previous authors have reported on patients who presented with pseudocyesis in the context of preexisting mood disorders, including major depressive disorder, bipolar disorder, schizophrenia, and schizoaffective disorder [7]. Our literature review revealed only two previous cases involving patients who developed pseudocyesis in the context of an acute manic episode [8, 9].

## 2. Case Presentation

We report on a 35-year-old female with a history of schizoaffective disorder, bipolar type, and cocaine use disorder, with multiple previous psychiatric admissions, who was brought to our hospital's crisis unit after her parents called police, complaining that she had become aggressive and was “acting delusional” during an argument. According to her parents, the patient had demanded a DNA test to prove that her parents were not her biological parents and that her son was not her biological son. One week prior to admission, the patient's parents had reported her missing when she “disappeared” from their home soon after being discharged from our hospital following a similar presentation. Prior to admission, the patient's medications included lamotrigine, clonazepam, and long-acting injectable aripiprazole; however, her adherence to treatment recommendations was poor.

Although her parents claimed that she resided with them and her 2-year-old son, the patient endorsed a recent history of homelessness lasting several months. The patient said that during this period of homelessness, she had been sexually assaulted by a stranger in his hotel room. Collateral revealed that per court order, the patient's continued custody of her son was contingent on her adherence to psychotropic medications and inpatient drug rehabilitation. The patient admitted to using recreational drugs frequently over the past 6 months, including crack cocaine, which she stated she had used during the 1-week interval between hospitalizations.

During her initial mental status examination, the patient was poorly groomed, uncooperative, intrusive, and irritable. Her speech was pressured, and she described her recent sleep as poor. She commented that she intended to become pregnant and to sell the baby to an agency for $6,000. She made religious statements about “being fruitful and multiplying” and admitted to recently engaging in hypersexual behavior, explaining that “I have boyfriends on the streets.” She underwent testing for sexually transmitted infections, with reassuring results. She declined voluntary inpatient psychiatric admission and was subsequently screened and committed. We discontinued clonazepam at the time of admission, and we restarted the patient on lamotrigine, adding oral aripiprazole to supplement her long-acting injectable aripiprazole.

During her hospitalization, the patient faced several barriers to efficient psychiatric stabilization. First among these was her insistence on refusing any medications that might cause weight gain, including olanzapine. She endorsed allergies to haloperidol, risperidone, lurasidone, and lithium, with symptoms such as “anxiety,” “eye rolling,” and “facial twitching” in response to these drugs. Because she was adherent to oral lamotrigine and aripiprazole, we chose not to seek treatment over objection to administer a stronger antipsychotic medication that would also serve as an effective mood stabilizer. On hospital Day 4, the patient appeared virtually in family court, where she received an ultimatum to cooperate with treatment recommendations in order to maintain custody of her son. Nonetheless, she remained adamant about not being placed on “weight gainers,” stating that “I can have nine more babies. I want to be sexy.” On hospital Day 6, she tested positive for COVID-19 and was quarantined for 10 days on a medical floor. Her respiratory symptoms included cough and rhinorrhea, and she continued to display manic symptoms, including elated affect, hyperverbality, grandiosity, religious preoccupation, and inappropriate laughter. She exhibited behaviors suggestive of sexual preoccupation, such as raising her shirt during interviews to reveal a protuberant abdomen and repeatedly endorsing a desire to “stay sexy.”

After her quarantine, the patient returned to the psychiatric unit with her manic symptoms unresolved. On hospital Day 17, she requested a pregnancy test due to concerns that she might have become pregnant because she “had sex with a bunch of guys because I was homeless.” The patient seemed reassured when we explained that her admission pregnancy test was negative. We ordered a new quantitative *β*-hCG test, which was likewise negative. On Day 18, the patient received a maintenance dose of long-acting injectable aripiprazole. On Day 19, she complained of abdominal and back pain, stating “I've been in labor since last night.” She elaborated that “I can feel the head in my crotch” and “when I lay on my back, I feel the little hands and feet.” She also stated that her “water broke” and her “mucus plug broke.” Consistent with the diagnostic criteria for pseudocyesis, the patient exhibited several physical signs and symptoms of pregnancy, including a distended abdomen, intermittent “pulsating” bilateral lower abdominal cramps, and sensations of fetal movement. In addition, she endorsed amenorrhea for the past year, except for some “mild spotting” three weeks previously. Convinced that her two recent pregnancy tests were “false negatives” and frustrated that members of the treatment team did not believe she was pregnant, she threatened to sue the hospital for failing to provide obstetric care. The patient lamented that “you all think I'm crazy, but I'm actually pregnant.” At this time, she displayed an intensifying religious preoccupation and stated that she was able to communicate with angels and “see Jesus.” She proclaimed that she herself was a prophet who could interpret the “auras” of hospital staff.

The patient agreed to a pelvic exam, which revealed a distended, nontender abdomen. The uterus was nongravid. The patient received acetaminophen and heating pads, and we ordered a transabdominal ultrasound to better assess the etiology of her abdominal pain and in the hopes of resolving her pseudocyesis via additional evidence of nonpregnancy. At this time, the patient began to refuse all previously prescribed medications because she believed these would harm her “baby.” She did, however, agree to begin fluphenazine elixir, which we prescribed to address worsening psychosis.

When she arrived in the ultrasound suite, the patient offered the radiology technologist four million dollars “if you get this baby right.” She became euphoric while viewing the ultrasound monitor, exclaiming “That's a head, and that's a penis!” Her speech was pressured, and she perseverated on religious themes, requesting that all non-Christian staff leave the suite and instructing staff to “resist the Devil and he will flee from you!” She also demonstrated sexual preoccupation, recalling an experience in which she had “danced after I had sex” and exclaiming about the size of her “baby's penis,” predicting that he would be “sexy like his daddy.” After the procedure, the patient happily told fellow patients that she would soon deliver another child. She said, “I have been having contractions for 19 hr. When the results come back and they find out I am not crazy, they will finally send me to maternity.”

An on-call psychiatry resident presented the radiology report (which revealed no intrauterine gestational sac) to the patient on hospital Day 20. She emphatically refuted the report, articulating a persecutory delusion of a conspiracy waged against her by the treatment team. She stated that the radiologist must have been “paid off” to switch her ultrasound with that of another patient. She continued to refuse medications that she felt might harm her “baby”; however, she agreed to an increase in her dose of fluphenazine. Following this dose increase, her affect remained labile, with frequent swings between euphoria and irritability, but she ceased making comments indicative of religious and sexual preoccupation. As our hospital is a short-term care facility, we decided to refer the patient to a higher level of care facility. On the day of her discharge, the patient told a nurse that “I don't think I'm pregnant anymore; it's all the shit I have inside me,” referring to her constipated bowels.

## 3. Discussion

Our case exemplifies the multifaceted nature of pseudocyesis, a condition whose etiology, diagnostic classification, and ideal management all remain elusive. For the balance of this report, we shall discuss how the case sheds light on unanswered questions regarding the epidemiology, pathogenesis, diagnosis, and treatment of pseudocyesis.

Previous authors have propounded various theories about the pathogenesis of pseudocyesis. Azizi and Elyasi [2] categorized these hypotheses into three major camps: somatopsychic, psychosomatic, and psychophysiological. The somatopsychic theory proposes that pseudocyesis “can be initiated by a coincidental physiologic change,” such as an ovarian cyst or abdominal distension due to constipation [10]. A susceptible patient may misinterpret such physiologic changes as signs of pregnancy, triggering the development of somatic symptoms.

The psychosomatic theory suggests, with psychodynamic overtones, that pseudocyesis “begins with fantasies of pregnancy and leads to physiological symptoms” [2]. For example, a 2013 case report describes a 40-year-old Indian woman who developed the condition after her husband blamed her for “not having a male child for generational continuity and economic security in old age” [11]. The higher prevalence of pseudocyesis among women in developing countries and potentially among socioeconomically disadvantaged patients in developed countries lends some credence to the psychosomatic theory. Our patient's pronouncement about selling her unborn child to an agency for $6,000 demonstrates her belief that pregnancy might afford her a degree of enhanced financial security.

The psychophysiological theory of pseudocyesis relies on evidence that women with this condition commonly suffer from comorbid depressive disorders to assert that neuroendocrine imbalances could play a role in its pathogenesis. In their 2013 review, Tarin et al. [12] lean heavily on the monoamine hypothesis of depression in contending that “pseudocyetic women may have a deficit in brain dopamine activity.” They postulate that because dopamine is a GnRH inhibitor, pseudocyesis would be characterized by increased pulsatile secretion of GnRH, LH, and PRL, resulting in such downstream effects as oligomenorrhea and galactorrhea. The present case provides an interesting example of pseudocyesis possibly triggered by a neuroendocrine imbalance, as the patient developed the condition within days after contracting COVID-19 disease and less than 1 day after receiving an initial dose of the long-acting injectable formulation of the partial dopamine agonist aripiprazole. Although neuroimmunology researchers have just recently begun to characterize COVID-19-related neuroendocrine changes, there is some evidence that COVID-19 can induce an increase in prolactin levels [13], which might trigger pseudocyesis. Interestingly, aripiprazole is the sole antipsychotic medication associated with a reduction in serum prolactin levels [14], but the combined impact of COVID-19 and aripiprazole on prolactin levels has not been described. To complicate matters, aripiprazole's status as a partial D2 agonist has generated speculation that its use might worsen psychosis in susceptible patients [15]. Our patient's development of new psychotic symptoms, including paranoid and persecutory delusions, followed within hours of the administration of her maintenance dose of long-acting injectable aripiprazole, a presentation that echoes previous case reports of patients who relapsed into psychosis after initiation of aripiprazole [16]. Additional translational research is needed to elucidate the neuroendocrine hypothesis of pseudocyesis and to assess the robustness of any association between COVID-19 disease, aripiprazole, and exacerbation of psychosis.

Just as the pathogenesis of pseudocyesis remains opaque, its diagnostic classification and ideal management are characterized by much ambiguity. Pseudocyesis is an ancient condition, with 12 cases appearing in the Hippocratic Corpus [17]. Nonetheless, DSM authors have struggled to locate an appropriate diagnostic rubric in which to categorize it, perhaps because the condition glaringly defies the mind–body dichotomy embedded within conventional medical thought [18]. Our patient's belief that she was pregnant resolved soon after titration of fluphenazine, prompting us to consider whether pseudocyesis is an inherently psychotic condition meriting aggressive D2 receptor blockade. In contrast to our clinical experience, DSM-5 does not classify pseudocyesis as a psychotic disorder but lumps the condition under the new category “other specified somatic symptom and related disorders.” Previous writers have attempted to distinguish pseudocyesis from its close (and plainly psychotic) companion, the delusion of pregnancy, on the basis that delusion of pregnancy involves a “fixed belief of being pregnant but in the absence of physical signs and symptoms suggestive of pregnancy” [9]. However, as Seeman [5] comments in a 2014 case report, the theoretical line demarcating the two conditions is “blurred.”

DSM-5 introduced a new category of somatic symptoms and related disorders to replace the DSM-IV category of somatoform disorders, in which pseudocyesis was previously classified. DSM-5 describes DSM-IV nosology regarding somatoform disorders as “confusing” due to “a great deal of overlap across the somatoform disorders and a lack of clarity about the boundaries of diagnoses” [1]. DSM-IV authors opined that the common feature of all somatoform disorders is “the presence of physical symptoms that suggest a general medical condition,” which are nonetheless “not fully explained by a general medical condition” [19]. Despite classifying pseudocyesis under a fresh diagnostic heading, DSM-5's approach to the condition recapitulates DSM-IV's focus on medically unexplained symptoms. Indeed, DSM-5 acknowledges that “medically unexplained symptoms remain a key feature in conversion disorder and pseudocyesis” because in both conditions, “it is possible to demonstrate definitively…that the symptoms are not consistent with medical pathophysiology” [1]. Curiously, DSM-5 authors admit that “grounding a diagnosis on the absence of [a medical] explanation is problematic and reinforces mind–body dualism” [1]. Furthermore, they acknowledge that diagnoses such as pseudocyesis and conversion disorder have contributed to stigma, stating that “[p]erhaps because of the predominant focus on lack of medical explanation, individuals regarded these diagnoses as pejorative and demeaning, implying that their physical symptoms were not ‘real'” [1].

The present case throws into stark relief the DSM-5 authors' concerns about stigma, elevating such issues from the realm of mere academic supposition to the poignant reality of a patient's experience of shame, confusion, and alienation both before and after being confronted with medical evidence disproving her conviction that she was pregnant. Our patient repeatedly voiced distress over her feeling that the hospital staff thought she was “crazy.” This experience of stigma fueled her persecutory delusions about a “conspiracy” designed to falsely deny her pregnancy status.

As several previous authors have commented, there are no clinical guidelines or protocols regarding the management of pseudocyesis. Some have suggested that the lynchpin of its management “is to help these patients recognize the illness” by proving that they are not, in fact, pregnant [20]. In caring for our patients, we learned that delivering test results can be a precarious task, for if the messenger is perceived as judgmental or perfunctory, the therapeutic alliance may suffer irreversible damage. In approaching this exquisitely sensitive clinical scenario, we recommend delivering the news during a private interview with a clinician who has previously established a positive rapport with the patient. In appropriate cases, we would advocate for a staged approach in which a physician first informs the pseudocyetic patient that pregnancy is unlikely and then reassures her that further tests will be performed to fully rule out the possibility. Simultaneously, it is important to validate the patient's symptoms of pregnancy by providing adequate pain control, as well as an obstetric consultation to help alleviate her concerns for the well-being of her “child.”

## 4. Conclusions

Pseudocyesis is a complex psychiatric manifestation characterized by physical signs and symptoms resembling those of pregnancy. The condition's exact cause remains unclear, but it is important to acknowledge that pseudocyesis presents with genuine symptoms, which can create challenges in diagnosis and management. Because cases of pseudocyesis often involve complex patient care requirements, physicians must adopt a comprehensive and compassionate approach in order to preserve the therapeutic alliance and optimize patient outcomes.

In addition to providing suggestions for the sensitive management of pseudocyesis based on our clinical experience, this case report offers the following contributions to the existing literature on an ancient condition: (1) demographically, our case supports the hypothesis that socioeconomically disadvantaged patients living in developed countries may be especially vulnerable to pseudocyesis; (2) the timing of our patient's presentation in the context of COVID-19 illness and following initiation of a partial dopamine agonist highlights the possibility of a neuroendocrine mechanism for this condition; (3) we add to the literature another case of pseudocyesis arising during an acute manic episode, which has been reported only rarely in the past; (4) our case highlights the challenges DSM authors have faced in attempting to characterize pseudocyesis; (5) our patient's lived experience of shame and alienation in response to being told she was not pregnant recapitulates DSM authors' concern that a diagnosis of pseudocyesis may be perceived as pejorative and demeaning.

## Data Availability

The patient data used to support the findings of this study have not been made available because of the need to protect patient privacy.
